# Exploring the impact of arginine-supplemented immunonutrition on length of stay in the intensive care unit: A retrospective cross-sectional analysis

**DOI:** 10.1371/journal.pone.0302074

**Published:** 2024-04-26

**Authors:** Niels D. Martin, Laura L. Schott, Mary K. Miranowski, Amarsinh M. Desai, Cynthia C. Lowen, Zhun Cao, Krysmaru Araujo Torres

**Affiliations:** 1 Department of Surgery, Perelman School of Medicine, University of Pennsylvania, Philadelphia, Pennsylvania, United States of America; 2 PINC AI^™^ Applied Sciences, Applied Research, Premier Inc., Charlotte, North Carolina, United States of America; 3 Regulatory and Medical Affairs, Research and Development, Active and Medical Nutrition, Nestlé Health Science, Bridgewater Township, New Jersey, United States of America; 4 Market Access, Active and Medical Nutrition, Nestlé Health Science, Bridgewater Township, New Jersey, United States of America; 5 Regulatory and Medical Affairs, Research and Development, Nestlé Health Science, Bridgewater Township, New Jersey, United States of America; University of Asia Pacific, BANGLADESH

## Abstract

**Background:**

Arginine-supplemented enteral immunonutrition has been designed to optimize outcomes in critical care patients. Existing formulas may be isocaloric and isoproteic, yet differ in L-arginine content, energy distribution, and in source and amount of many other specialized ingredients. The individual contributions of each may be difficult to pinpoint; however, all cumulate in the body’s response to illness and injury. The study objective was to compare health outcomes between different immunonutrition formulas.

**Methods:**

Real-world data from October 2015 –February 2019 in the PINC AI^™^ Healthcare Database (formerly the Premier Healthcare Database) was reviewed for patients with an intensive care unit (ICU) stay and ≥3 days exclusive use of either higher L-arginine formula (HAF), or lower L-arginine formula (LAF). Multivariable generalized linear model regression was used to check associations between formulas and ICU length of stay.

**Results:**

3,284 patients (74.5% surgical) were included from 21 hospitals, with 2,525 receiving HAF and 759 LAF. Inpatient mortality (19.4%) and surgical site infections (6.2%) were similar across groups. Median hospital stay of 17 days (IQR: 16) did not differ by immunonutrition formula. Median ICU stay was shorter for patients receiving HAF compared to LAF (10 vs 12 days; *P*<0.001). After adjusting for demographics, visit, severity of illness, and other clinical characteristics, associated regression-adjusted ICU length of stay for patients in the HAF group was 11% shorter [0.89 (95% CI: 0.84, 0.94; *P*<0.001)] compared to patients in the LAF group. Estimated adjusted mean ICU length of stay was 9.4 days (95% CI: 8.9, 10.0 days) for the HAF group compared to 10.6 days (95% CI: 9.9, 11.3 days) for the LAF group (*P*<0.001).

**Conclusions:**

Despite formulas being isocaloric and isoproteic, HAF use was associated with significantly reduced ICU length of stay, compared to LAF. Higher arginine immunonutrition formula may play a role in improving health outcomes in primarily surgical critically ill patients.

## Introduction

It is estimated that >25% of all hospital stays include an intensive care unit (ICU) admission [1]. Patients admitted to an ICU experience a 13% higher rate of in-hospital mortality, 15% higher rate of readmission, 8% higher rate of post-discharge emergency department visits, and 8% lower 1-year post-discharge survival rate compared to non-ICU patients or the general population [2, 3]. Critical illness and ICU admission are associated with malnutrition, and malnutrition correlates with poor clinical outcomes, and increased hospital costs, length of stay (LOS), and readmission [4–8]. Nutritional intervention is essential for surgical and ICU patients [9].

Enteral nutrition (EN) is advised for critically ill patients in American and European intensive care and clinical nutrition society guidelines [10–14]. Benefits of EN include maintenance of gut integrity, modulation of the systemic immune response, and the attenuation of disease severity [11]. Immunonutrition enteral formulas, which include specialized nutrients to modulate the body’s response to illness and injury, may be beneficial for surgical patients and those in the ICU [11, 15–21]. The literature suggests that for surgical and trauma patients, high-protein EN formulas containing supplemental arginine and other immunonutrients are associated with better outcomes, including decreased LOS and reduced infections [11, 22–24]. A meta-analysis examining immunonutrition versus standard nutrition formulas in surgical cancer patients found reduced risk of infections, including a sub-analysis of multiagent versus single-agent immunonutrition where the combination of arginine, nucleotides, and n-3 fatty acids significantly reduced the risk of wound and respiratory infections (by 36% and 39%, respectively) [20]. Other nutrients may also play a role in mitigating the immune response [25, 26].

Recent work showed that critically ill patients who received standard high-protein EN were older, were less likely to be surgical or trauma patients, had shorter LOS, and had higher total cost per day in adjusted analyses compared to patients that received arginine-supplemented enteral immunonutrition [27]. These findings suggest the need for further exploration specific to immunonutrition formulas. Although similarly designed for nutrition and tolerance in critically ill patients, immunonutrition formulas differ in amount and type of immunonutrients, i.e., arginine and other micro and macronutrients [17, 27, 28]. Research directly comparing commonly used immunonutrition formulas and clinical outcomes is sparse, and additional research on immunonutrition formulas in critically ill patients is recommended [10, 29]. This study used real-world data in a heterogeneous patient cohort from multiple US hospitals to examine differences in association between ICU LOS and the receipt of different isocaloric and isoproteic immunonutrition formulas containing either higher or lower amounts of L-arginine and other ingredient differences.

## Materials and methods

### Sample and study design

The sample consisted of inpatients age 18 years and older, discharged between October 2015 and February 2019, with a billing record of at least 3 days of use within 5 consecutive days of either higher L-arginine (18.7 g/L) formula (HAF), or lower L-arginine (11 g/L) formula (LAF), and a minimum of 1 billed day of ICU utilization. Both formulas contained equivalent amounts of protein and calories per liter (Table 1). A retrospective, cross-sectional study was conducted using data from the PINC AI^™^ Healthcare Database (formerly the Premier Healthcare Database) [30]. Immunonutrition formula type (i.e., HAF, LAF) was the exposure variable; ICU LOS was the primary outcome, and hospital LOS and surgical site infection were secondary outcomes. Presence of surgical site infection was determined via Medicare Severity Diagnosis Related Group (MS-DRG) at discharge.

**Table 1:** Nutrition comparison of the immunonutrition formulas (per liter).

EN Formula Contents	HAF	LAF
**Kcal/mL**	1.5	1.5
**Protein, g (%)**	94 (25)	93.8 (25)
**Source**	hydrolyzed casein and arginine	hydrolyzed casein, whey, and arginine
**Supplemental L-arginine, g**	18.7	11
**Total arginine, g**	20.8	13
**Carbohydrate, g (%)**	140 (38)	172.4 (45)
**Fiber, g**	--	7.5
**Fat, g (%)**	63.6 (37)	51 (31)
**Ω6:Ω3**	1.5:1	1.7:1
**EPA + DHA, g**	4.9	3.7
**MCT:LCT**	50:50	20:80
**MCT, g**	31.8	10.2
**Supplemental nucleotides, g**	1.8	--
**Select micronutrients**		
**Vitamin C, mg**	1000	304
**Selenium, mcg**	100	78
**Zinc, mg**	36	30.8

DHA, docosahexaenoic acid; EN, enteral nutrition; EPA, eicosapentaenoic acid; HAF, higher L-arginine (Impact^®^ Peptide 1.5, Société des Produits Nestlé S.A) formula; LAF, lower L-arginine (Pivot^®^ 1.5, Abbott Laboratories) formula; LCT, long-chain triglycerides; MCT, medium-chain triglycerides; Ω6:Ω3, ratio of omega 6 and omega 3 fatty acids

Data were from an all-payer, geographically diverse, hospital-based database representative of US hospitals, capturing service and billing information from all therapeutic areas for approximately 25% of all inpatient discharges in the country. The database serves as a reliable source used by national healthcare agencies, life science companies, and academic institutions [30]. Data consist of a nonrandom sample and are subject to limitations of an administrative database, including accuracy of coding and absence of certain clinical details. The database has been certified as de-identified and is not considered human subjects research. Study data and recorded information could not be identified directly or through identifiers linked to individuals. The authors accessed the data for research purposes on March 1, 2020, and could not identify individual participants during or after data collection. All data were compliant with the Health Insurance Portability and Accountability Act (HIPAA). As a result of these factors and US federal regulation 45 CFR 46, the study was deemed exempt from institutional review board evaluation and informed consent [31]. The study followed Strengthening the Reporting of Observational Studies in Epidemiology (STROBE) guidelines [32].

### Baseline characteristics

Demographic information included patient age, self-reported sex, race, and ethnicity, and primary insurance payer. Clinical characteristics captured at discharge via ICD-10-CM and billing codes included trauma status, mechanical ventilation, rectal tube, diarrhea, constipation, and wound dehiscence/disruption; and via MS-DRG codes were surgery status, and extracorporeal membrane oxygenation (ECMO) or tracheostomy procedure. Severity of illness and risk of mortality were assessed via the 3M^™^ All Patient Refined DRG (APR DRG) Classification System (i.e., minor, moderate, major, or extreme). Elixhauser comorbidity score (overall) and each comorbidity assessed were evaluated at discharge via ICD-10-CM codes [33, 34]. Additional comorbidities included malnutrition, pneumonia, septicemia, urinary tract infection, and *C*. *difficile* infection. Nutrition product utilization tracked total days used, EN pattern (consecutive vs not) and volume per day of feeding. Medication use evaluated prescription and number of days for antidiarrheal, antiemetic, and antibiotic class drugs. Visit characteristics included attending physician specialty, and admission type, point of origin, and discharge status, as submitted by hospitals according to Centers for Medicare and Medicaid Services criteria. Hospital characteristics included US census region (i.e., Midwest, Northeast, South, West), teaching status, urban/rural location, and bed size (i.e., <500 beds vs ≥500) of the facility where the patient was hospitalized.

### Statistical analyses

The hypothesis was that ICU LOS would differ for patients receiving HAF and LAF. Comparisons between immunonutrition groups were done via Chi-square tests for dichotomous and categorical variables, which were reported as n (%). Continuous variables were reported as median (interquartile range, IQR) or mean (standard deviation, SD), and comparisons were made via Wilcoxon rank sum or t-tests, respectively. Prior to initiation, the study was powered at 80% and alpha value = 0.05 to detect a 2-day difference in ICU LOS, with a minimum sample size of 636 in each group. To minimize potential biases, hypotheses were established a priori, and design, analysis, and publication of the study were not contingent on the sponsor’s approval or censorship.

A multivariable generalized linear model with negative binomial variance and log link function was used to evaluate associations between immunonutrition group and ICU LOS. For ease of interpretation, regression coefficients and 95% confidence intervals (CI) were exponentiated. Due to the log transformation, the exponentiated coefficients were interpreted as the percentage difference between the groups. In addition, least square means were used to estimate the adjusted mean ICU LOS for each group. Final covariates, chosen from clinically and statistically relevant variables, included patient demographics (i.e., age category, sex, and race), and visit (i.e., insurance payer, admission type, and discharge status), hospital (i.e., size and region), and clinical characteristics (i.e., APR DRG severity of illness and risk of mortality, surgery status, trauma status, EN pattern, nutrition units billed per day, septicemia, pneumonia, obesity, congestive heart failure, cancer, complicated diabetes, renal failure, malnutrition, ECMO or tracheostomy, mechanical ventilation, rectal tube, days of antidiarrheal medication use, and wound dehiscence/disruption). To assess model fit and properties of variables in the model, sensitivity analyses were completed. All analyses were conducted using SAS version 9.4. Statistical significance was defined as *P*<0.05.

## Results

### Sample characteristics

During the 3.5-year study period, 3,284 patients meeting inclusion criteria across 21 hospitals were identified (2,525 HAF, 759 LAF; Table 2). Mean age was 56.6 years (SD: 17.7). The sample was primarily male (67.5%), white (86.0%), and non-Hispanic/Latino or unknown ethnicity (97.3%). Primary insurance payer was Medicare (38.0%) or Medicaid (25.6%), followed by commercial (19.1%), managed care (8.1%), and other insurance (9.1%). Most patients were admitted from home (74.8%) and non-electively (88.0%). One-fifth of all study patients died during hospitalization, 22.3% were discharged home, and 47.2% were discharged to a long-term care or skilled nursing facility. Two-thirds of attending physicians were from a surgical specialty (66.9%). Comparisons between immunonutrition groups revealed differences in baseline characteristics. Patients receiving HAF had a higher frequency of being treated in a teaching hospital located in the Midwest/West, whereas patients receiving LAF had a higher frequency of being treated at a hospital in an urban setting, with 500+ beds, located in the Northeast/South (all *P*<0.001).

**Table 2:** Patient characteristics.

Characteristic	Total N = 3,284 n (%)	HAF N = 2,525 n (%)	LAF N = 759 n (%)
**Age, years, median (IQR)**	59 (25)	59 (24)	58 (28)
**Age group, years** *			
** 18–34**	492 (15.0)	357 (14.1)	135 (17.8)
** 35–49**	509 (15.5)	380 (15.0)	129 (17.0)
** 50–64**	1,060 (32.3)	836 (33.1)	224 (29.5)
** 65–79**	953 (29.0)	745 (29.5)	208 (27.4)
** ≥ 80**	270 (8.2)	207 (8.2)	63 (8.3)
**Female** **	1,068 (32.5)	866 (34.3)	202 (26.6)
**Race** **			
** White**	2,825 (86.0)	2,219 (87.9)	606 (79.8)
** Black**	244 (7.4)	151 (6.0)	93 (12.3)
** Other/unknown**	215 (6.5)	155 (6.1)	60 (7.9)
**Hispanic or Latino ethnicity** **	89 (2.7)	71 (2.8)	18 (2.4)
**Discharge status** **			
** Inpatient mortality**	636 (19.4)	484 (19.2)	152 (20.0)
** Home / Home healthcare**	733 (22.3)	620 (24.6)	113 (14.9)
** LTC/SNF/ICF/Rehabilitation**	1,547 (47.2)	1,122 (44.4)	425 (56.0)
** Other/Unknown**	368 (11.2)	299 (11.8)	69 (9.1)
**EN pattern of 3 consecutive days (versus EN for 3 days in 5)** *	2,938 (89.5)	2237 (88.6)	701 (92.4)
**Nutrition utilization, median (IQR)**			
** Days of EN use**	7 (8)	7 (7)	7 (7)
** Total 1000 mL units of EN billed** *	8.8 (10)	9 (10)	8 (9)
** EN units billed per day** **	1.18 (0.40)	1.20 (0.50)	1.17 (0.29)
**ECMO or tracheostomy**	833 (25.4)	641 (25.4)	192 (25.3)
**Mechanical ventilation** **	2,575 (78.4)	1,930 (76.4)	645 (85.0)
**APR DRG severity of illness**			
** Minor/moderate**	418 (12.7)	322 (12.8)	98 (12.7)
** Major /extreme**	2,866 (87.3)	2,203 (87.3)	663 (87.4)
**APR DRG risk of mortality** **			
** Minor/moderate**	644 (19.6)	531 (21.0)	113 (14.9)
** Major/extreme**	2,640 (80.4)	1,994 (79.0)	646 (85.1)
**Elixhauser comorbidity** ^a^ score, median (IQR)**	5 (4)	5 (4)	6 (5)
**Urinary tract infection**	444 (13.5)	335 (13.3)	109 (14.4)
**C. difficile infection**	161 (4.9)	116 (4.6)	45 (5.9)
**Wound dehiscence/disruption** *	101 (3.1)	86 (3.4)	15 (2.0)

APR DRG, All Patient Refined Diagnosis Related Groups Classification System; ECMO, extracorporeal membrane oxygenation; EN, enteral nutrition; HAF, higher L-arginine (18.7 g/L) formula; ICF, intermediate care facility; IQR, interquartile range; LAF, lower L-arginine (11 g/L) formula; LTC, long-term care facility; SNF, skilled nursing facility

* *P* < 0.05,

** *P* < 0.001

^a^ Elixhauser comorbidity score was assessed using Quan’s algorithm of primary or secondary ICD-10-CM diagnosis codes at discharge. Prevalence of each comorbidity was evaluated, including congestive heart failure, cardiac arrhythmias, valvular disease, pulmonary circulation disorders, peripheral vascular disorders, hypertension, paralysis, other neurological disorders, chronic pulmonary disease, complicated diabetes, uncomplicated diabetes, hypothyroidism, renal failure, liver diseases, peptic ulcer disease excluding bleeding, HIV disease (human immunodeficiency virus disease), lymphoma, metastatic cancer, solid tumor without metastasis, rheumatoid arthritis/collagen vascular disease, coagulopathy, obesity, weight loss, fluid and electrolyte disorders, blood loss anemia, deficiency anemia, alcohol abuse, drug abuse, psychosis, and depression.

### Clinical characteristics

Median days of feeding across groups was 7 days (IQR: 8). Total median volume of units billed was 1 unit higher for patients who received HAF compared to LAF, equating to a median daily difference of 30 mL (each *P*<0.01). Overall, 89.5% of patients received EN for 3 or more consecutive days. Prevalence of diarrhea was <5% and did not differ by EN formula. Prevalence of constipation was higher for patients receiving HAF (10.8%) compared to LAF (5.0%, *P*<0.001), whereas rectal tube use was lower (9.1% vs 19.4%, *P*<0.001, respectively). Across groups, prescription of antiemetic medications was 69.1% for a median of 2 days (IQR: 4). Patients who received HAF had a higher rate of antidiarrheal medication use compared to LAF (26.7% vs 15.7%, *P*<0.001) and for patients prescribed antidiarrheals, a higher median number of medication days (9 vs 3, *P*<0.001). A quarter of all patients had a MS-DRG coding of ECMO or tracheostomy, of which <1% received ECMO. Use of mechanical ventilation was reported 8.6% less frequently in patients receiving HAF compared to LAF (*P*<0.001). Wound dehiscence/disruption was noted in 3.1% of all patients.

**Fig 1:**
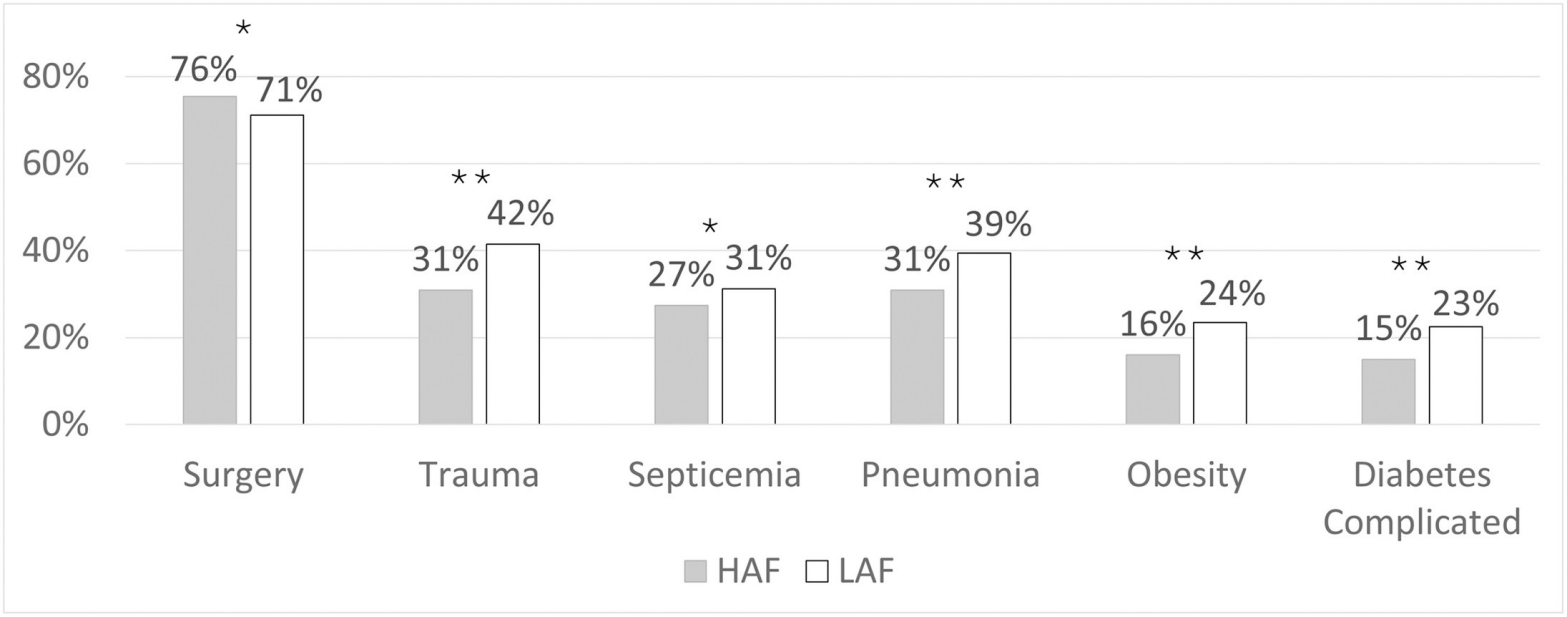
Clinical characteristics and comorbidities at discharge by immunonutrition formula group. HAF, higher L-arginine (18.7 g/L) formula; LAF, lower L-arginine (11 g/L) formula. * *P* < 0.05, ** *P* < 0.001.

Diagnosis of malnutrition did not differ between groups (28.9% overall), whereas prevalence of obesity was 7.5% lower for the HAF compared to LAF group (*P*<0.001) (Fig 1). Seventy-five percent of all patients had a surgical MS-DRG, and 33.6% had a trauma diagnosis. More patients in the HAF group were classified as surgical (difference 4.3%, *P* = 0.02), whereas more patients in the LAF group had a trauma diagnosis (difference 10.3%, *P*<0.001).

Overall, most patients were classified as major or extreme severity of illness (87.3%) and risk of mortality (80.4%), with the latter being 6% less frequent in the HAF cohort (*P*<0.001). Median Elixhauser comorbidity score was 1 point lower in the HAF cohort (*P*<0.001). Several specific comorbidities differed by immunonutrition cohort. Patients in the HAF cohort more frequently had cancer compared to the LAF cohort (difference 8.6%, *P*<0.001) but a lower frequency of neurological disorders, coagulopathy, or cardiac arrhythmia (differences ≥10%, all *P*<0.001) as well as pneumonia, septicemia, complicated diabetes, complicated hypertension, congestive heart failure, valvular disease, peripheral vascular disorders, renal failure, pulmonary circulation disorders, and paralysis (differences 3–9%, all *P*<0.05). Immunonutrition groups did not differ in proportion of patients with chronic pulmonary disease (23.4% overall), depression (17.4%), or liver disease (12.7%) comorbidities.

### Outcomes

**Fig 2:**
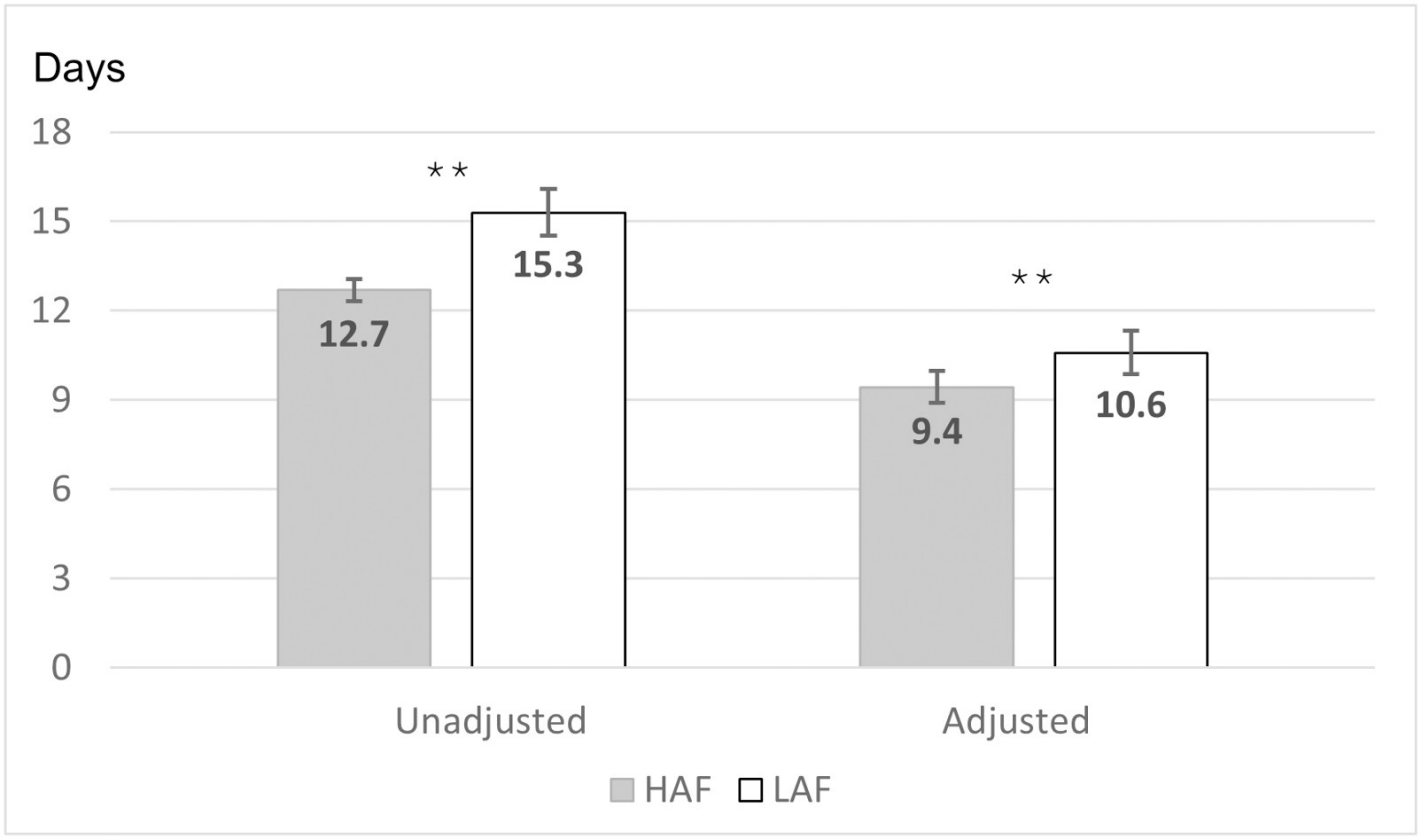
Mean ICU Length of Stay (days) by immunonutrition formula group ^a^. HAF, higher L-arginine (18.7 g/L) formula; ICU, intensive care unit; LAF, lower L-arginine (11 g/L) formula. ** *P* < 0.001. ^a^ Error bars are 95% confidence intervals for mean. Adjusted mean value estimated via general linear model least squares means function. Adjusted model included the following covariates: (i.e., age category, sex, race, primary insurance payer, admission type, discharge status, hospital bed size and geographic region, EN pattern, nutrition units billed per day, severity of illness, risk of mortality, surgery status, trauma status, septicemia, pneumonia, obesity, congestive heart failure, cancer, complicated diabetes, renal failure, malnutrition, ECMO or tracheostomy, mechanical ventilation, rectal tube, days of antidiarrheal medication use, and wound dehiscence/disruption.

**Fig 3:**
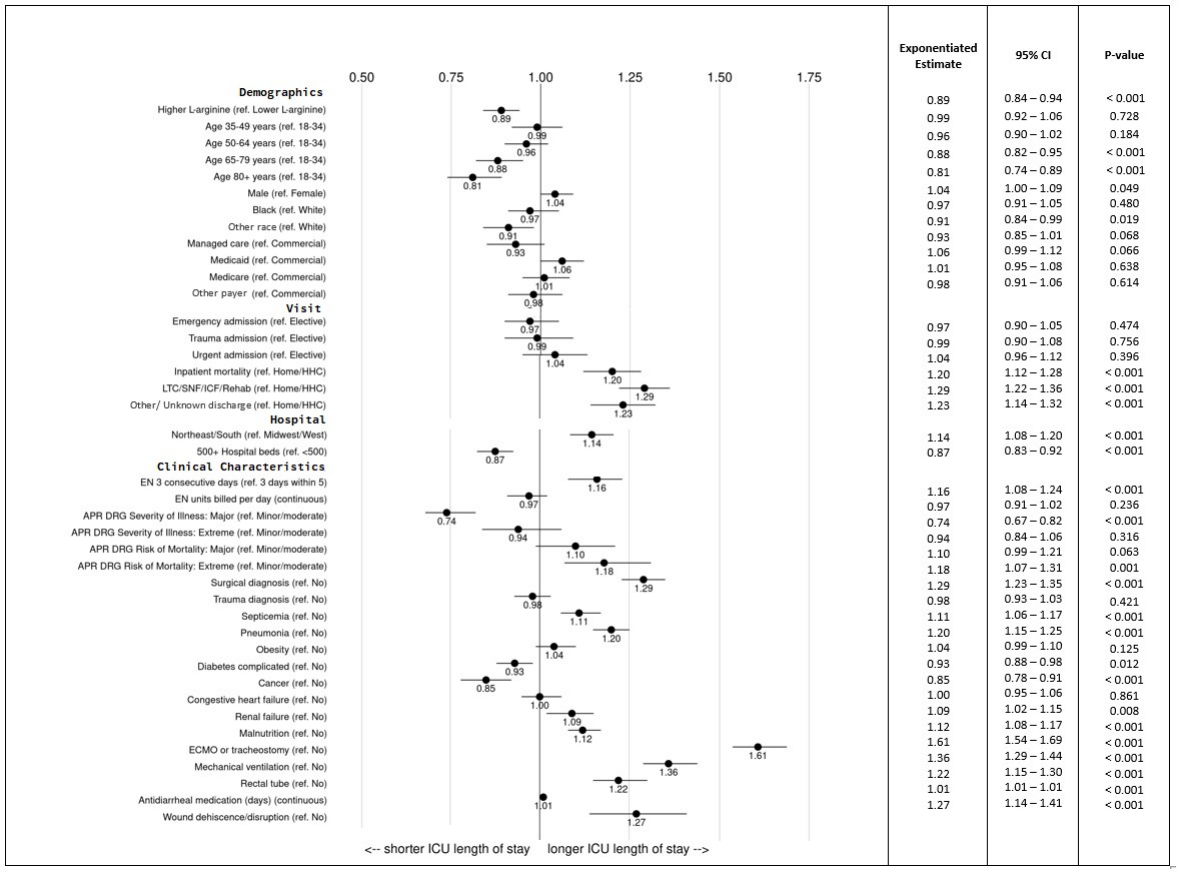
Multivariate regression estimates for ICU length of stay ^a^. APR DRG, All Patient Refined Diagnosis Related Groups Classification System; ECMO, extracorporeal membrane oxygenation; EN, enteral nutrition; ICF, intermediate care facility; ICU, intensive care unit; LTC, long-term care facility; ref. = reference group/category; SNF, skilled nursing facility. ^a^ After adjustment for covariates, regression-adjusted ICU length of stay was 11% shorter for patients receiving higher L-arginine (18.7 g/L) formula compare with patients receiving lower L-arginine (11 g/L) formula.

Mean ICU LOS was 2.6 days shorter for the HAF cohort compared to the LAF cohort, and the estimated adjusted mean difference was 1.2 days shorter per least square means in the multivariable model (*P*<0.001) (Table 3, Fig 2). In accordance, regression-adjusted ICU LOS was 11% shorter for patients in the HAF group compared to patients in the LAF group (*P*<0.001), after adjusting for multiple patient, visit, severity of illness, and other clinical characteristics, including inpatient mortality (Fig 3). Shorter ICU LOS was also associated with older age category, large hospital size, major APR-DRG severity of illness, and cancer (*P*<0.001).

**Table 3:** Unadjusted data.

Characteristic	Total N = 3,284	HAF N = 2,525	LAF N = 759
**ICU LOS, mean (SD)** **	13.3 (11.1)	12.7 (11.0)	15.3 (11.1)
**ICU LOS, median (IQR)** **	10 (11)	10 (11)	12 (11)
**Hospital LOS, median (IQR)**	17 (16)	17 (16)	18 (16)
**LOS live discharges, median (IQR)**	19 (17)	18 (17)	19 (17)
**Surgical site infections, n (%)**	203 (6.2)	151 (6.0)	52 (6.9)
**Percent of patients with antibiotic use** *	94.9	94.2	97.1
** Days of antibiotic use, median (IQR)**	19 (16)	19 (17)	19 (16)

ICU, intensive care unit; IQR, interquartile range; HAF, higher L-arginine (18.7 g/L) formula; IQR, interquartile range; LAF, lower L-arginine (11 g/L) formula; LOS, length of stay; SD, standard deviation

* *P* < 0.05,

** *P* < 0.001

Longer ICU LOS was associated with several characteristics in the multivariable model. For example, use of ECMO or tracheostomy and mechanical ventilation were associated with a 61% and 36% longer ICU LOS, respectively. Surgery diagnosis, evidence of wound dehiscence/disruption, not being discharged home, rectal tube, and pneumonia were associated with 20–30% longer ICU LOS in the adjusted model.

Surgical site infections were noted in 6.2% of patients and were similar across groups. Antibiotics were prescribed to 94.9% of patients for a median of 19 days (IQR: 16). Overall, median hospital LOS was similar between immunonutrition groups.

## Discussion

This study used real-world data to compare critical care patients, primarily surgical, receiving different immunonutrition formulas. After controlling for differences in demographic, visit, and clinical characteristics, on average, patients receiving HAF spent 1 day less in the ICU than patients receiving LAF. In univariate analysis, frequency of wound dehiscence/disruption and surgical site infections were low and clinically similar between cohorts.

The importance of nutritional assessment and protocols in the ICU and after surgery is well-documented [6, 8, 9, 14]. Nutrition intervention has been shown to reduce LOS, readmissions, and costs and improve survival [9, 35–37]. ICU LOS in our study was similar to the 13–14 days reported elsewhere for patients receiving immunonutrition [15, 23]. As expected, several covariates in the current analysis showed a stronger association with ICU LOS than immunonutrition, including risk of mortality, tracheostomy, mechanical ventilation, surgical diagnosis, wound dehiscence/disruption, rectal tube, and pneumonia. Nonetheless, HAF immunonutrition was significantly associated with shorter ICU LOS.

Four out of 5 study patients were categorized as major or extreme severity of illness and risk of mortality; however, only 28.9% of patients had a diagnosis of malnutrition, most likely due to variation in coding. Given data showing 49% of patients hospitalized >7 days remain malnourished or decline [38], malnutrition rate was expected much higher in our sample. The median 7 days study patients received HAF or LAF is comparable to an 8–9 day mean observed in a multi-center trial comparing immunonutrition containing L-arginine, n-3 fatty acids, and nucleotides with standard formula [15]. Furthermore, 5 days has been suggested as the minimum number of days for immunonutrition to be efficacious [39].

The potential for nutritional modulation of the immune response supports the use of immunonutrition formulas for patients undergoing major surgery or suffering extensive injury [11, 17, 22]. Prior research suggests immunonutrition formulas show benefit versus standard EN in patients having major elective surgery, and in critically ill surgical and trauma patients [11, 15, 16, 20–24, 27, 40, 41]. Accordingly, 74.5% of patients in the current study had a surgical diagnosis across a range of conditions. Results of many studies report that immunonutrition is associated with shorter LOS and fewer postoperative infectious or other complications [16, 22, 24, 42]. Mean LOS of 19–23 days reported in several studies was similar to hospital LOS found in both groups here, although other studies reported both shorter and longer stays (10–16 and 25–28 days) [15, 22, 24]. Rate of surgical site infection (6.2%) in the current study was similar to prior studies, although the range is large (4–63%) [15, 22, 24]. An a priori sub-study in a meta-analysis of immunonutrition in major elective surgery found a significant reduction in risk of infectious complications for studies comparing formulas containing a blend of arginine, n-3 fatty acids, and nucleotides to standard EN versus other arginine-supplemented EN compared to standard [22]. Differences in study populations, comparator groups, infection types, timing, and immunonutrient content of formulas likely influenced variation in results.

Although a definitive effect cannot be assigned to single ingredients in immunonutrition formulas, research suggests that certain nutrients are associated with particular clinical functions and potential benefits [17, 18, 25, 28, 43, 44]. For critically ill surgical and trauma patients, nutrition guidelines suggest administration of formulas to meet higher protein needs that also provide adequate energy and contain L-arginine and fish oil [11]. European guidelines on surgical nutrition also include nucleotides on the list of EN immunonutrients suggested for malnourished patients having major cancer surgery [45].

Arginine serves as a conditionally essential amino acid, participating in fundamental metabolic pathways [43, 44, 46]. The role of arginine in immune function modulation, nitric oxide synthesis, and wound healing may be particularly advantageous for critically ill surgical patients [18, 28, 43, 46]. The immunosuppression that commonly follows major surgery is linked to the depletion of arginine by myeloid deprived suppressor cells expressing arginase-1, and thereby causing T-lymphocyte dysfunction [47, 48].

It has been estimated that patients receiving enteral immunonutrition in the ICU receive on average 15 to 30 g of supplemental L-arginine per day [28]. A randomized controlled trial comparing different daily doses of L-arginine (5.7 vs 12.3 vs 18.9 g) postoperatively in enterally fed head and neck cancer patients found LOS and fistula formation were minimized most in the group receiving the highest dose [49]. In the current study, all patients received about 1 liter of formula per day with the difference in supplemental L-arginine being approximately 7.7g/L between HAF and LAF.

In addition to the difference in L-arginine content, the HAF examined here contains a higher amount of omega-3 fatty acids than LAF, as well as supplemental nucleotides. Omega-3 fatty acids and eicosapentaenoic acid (EPA) and docosahexaenoic acid (DHA) from fish oil have anti-inflammatory actions and may help mitigate arginine deficiency by producing less inflammatory prostaglandins, thereby reducing induction of arginase-1 [50, 51]. Nucleotides support replication of rapidly dividing cells (i.e., lymphocytes) by providing a source of purine and pyrimidine bases for DNA/RNA production, and in pre-clinical studies have been shown to help clear pathogens through the action of macrophages and natural killer cells [20, 52]. In particular, surgical stress or episodes of infection following injury show an increased demand for nucleotides to synthesize immune cells, for tissue repair, and to maintain organ function; however, there are very little data studying nucleotides in adults separate from immunonutrition formulas [52].

In association with immune dysfunction, inflammation, and oxidative stress, the body’s response to critical illness and injury involves trace elements and select vitamins. As such, immunonutrition formulas often include higher amounts of zinc, selenium, and vitamin C. Guidelines have identified these micronutrients, among others, for higher risk of deficiency during critical illness as well as inadequacy associated with worse outcomes [26]. Both LAF and HAF have increased amounts of vitamin C, selenium, and zinc compared to standard formula. Given the variability in nutrients and dosing of immunonutrition diets, guidelines suggest additional evaluation of specialized nutrition formulas [10].

Study limitations reflecting our use of observational administrative data include reliance upon diagnoses and procedure coding (e.g., codes do not differentiate between diagnoses on admission vs clinical outcomes). Further, because the study did not include chart review, the administration, adequacy, and timing of nutrition provided, as well as the total dose of antidiarrheals and the magnitude and severity of diarrhea could not be accounted for. Whether patients received additional supplementation of certain micronutrients outside of an EN formula was also not assessed. Although many potential confounders were included in adjusted analyses, distinctions between immunonutrition cohorts, including lack of hospital overlap, were evident. Differences in unmeasured characteristics may exist and could have influenced outcomes. Because authors were employees or received direct or indirect payment from the study sponsor for this work, potential biases were minimized by presenting both significant and non-significant results.

The importance of maximizing nutritional benefits in critically ill patients is validated by the associations between ICU stay, nutritional status, and clinical and healthcare utilization outcomes. Given that charges from ICU services may represent nearly 50% of aggregate total hospital charges [1], the decrease in ICU LOS measured in the present study helps explain earlier work showing HAF associated with a lower hospital cost per day than either LAF or standard high-protein EN [27]. In the US, 5 million patients annually are admitted to the ICU at an average of $4,300 per day [53]. Given the average charge per day outside the ICU is $1,143 [54], the savings estimated from one less day in the ICU are significant. From a clinical standpoint, ICUs admirably provide lifesaving care for critically ill patients. Nonetheless, the risk of a hospital-acquired infection or a complication caused by immobility or medication error increases the longer a patient is in the ICU [55, 56].

## Conclusions

In sum, higher L-arginine-supplemented immunonutrition also containing fish oil and nucleotides may play a role in improving health outcomes in the critically ill as evidenced by shorter ICU LOS, primarily in surgical patients. In this study of administrative data, immunonutrition formula type was not associated with significant unadjusted differences in hospital stay, surgical site infections, or mortality. The reduction in ICU LOS begets further questions about other clinical outcomes relevant to immunonutrition and ICU LOS. Possible candidates for future study include post-admission sepsis, pneumonia, and surgical site infection, although each would require extensive analysis involving chart review. Using a select immunonutrition formula for patients in the ICU may provide healthcare utilization savings, nonetheless, given the heterogeneity of patients, diagnoses, and phase of illness, individual circumstances should guide nutrition intervention for patients.
